# A FABulous approach to image human macrophages

**DOI:** 10.1007/s00259-026-08180-y

**Published:** 2026-09-28

**Authors:** Borna Roohani, Ayla Vaughn Embs, Robin Kumar, Supum Lee, Samantha R. Katz, Chloe La Prairie, Dajah Nash, Mann Dangarwala, Harriet M. Kluger, Daryl E. Klein, Ping Zhang, Benjamin M. Larimer, Bryce Nelson, Bernadette Marquez-Nostra

**Affiliations:** 1 Department of Radiology, University of Alabama at Birmingham, 1824 6th Ave. S., Wallace Tumor Institute 310C, Birmingham, AL 35233 USA; 2 Department of Diagnostic and Interventional Radiology and Nuclear Medicine, University Medical Center Hamburg-Eppendorf, Hamburg, Germany; 3 Department of Radiology and Biomedical Imaging, Yale PET Center, Yale University, New Haven, CT USA; 4 Medical College of Georgia, Augusta, GA USA; 5 Department of Pediatric Dentistry, University of Alabama at Birmingham, Birmingham, AL USA; 6 Department of Medicine, Yale University, New Haven, CT USA; 7 Department of Pharmacology, Yale University, New Haven, CT USA; 8 Orion Pharma, Cambridge, UK

**Keywords:** CD68, Macrophages, ImmunoPET, Phage display, Fab

## Abstract

**Rationale:**

Imaging tumor-associated macrophages (TAMs) may reflect prognosis, therapeutic response and radiotheranostic potential. We report the discovery and development of a synthetic human Fab as the first immunoPET tracer targeting human CD68 (huCD68), the gold standard histopathological biomarker of pan-macrophages.

**Methods:**

Phage display was used to identify novel huCD68-specific Fabs. The lead Fab for immunoPET development was chosen based on expression yield and stability. Fab was conjugated to p-SCN-Bn-DFO and radiolabeled with ^89^Zr, yielding [^89^Zr]Zr-DFO-Fab. Stability and specific binding of the tracer to THP-1 macrophages were assessed in vitro. The specificity of the tracer in vivo was characterized by PET imaging of two different animal models: 1) BALB/c mice bearing huCD68 antigen- or irrelevant protein-plugs, and 2) tumor xenografted NSG mice intratumorally injected with either THP-1 macrophages or THP-1 undifferentiated cells. Finally, tumors were subjected to immunofluorescence (IF) staining of huCD68 and correlated with PET standardized uptake values (SUV).

**Results:**

Four novel huCD68-specific Fabs were identified. Fab8 was chosen as the lead based on high expression yield, purity, and stability. In vivo, [^89^Zr]Zr-DFO-Fab8 uptake was significantly higher in huCD68 antigen-plugs than in irrelevant protein-plugs at 1- and 4-h post-injection (p.i.). Tracer uptake in THP-1 macrophage tumors was also significantly higher than in THP-1 undifferentiated tumors at 1- and 6-h p.i.. IF of tumors confirmed higher density of huCD68 in those implanted with THP-1 macrophages than those with THP-1 undifferentiated cells. CD68 expression highly correlated with immunoPET SUV.

**Conclusion:**

[^89^Zr]Zr-DFO-Fab8 binds specifically to huCD68, supporting the feasibility of direct PET-to-histopathogical correlation of total TAM burden.

**Supplementary Information:**

The online version contains supplementary material available at https://doi.org/10.1007/s00259-026-08180-y.

## Introduction

Macrophages are versatile immune cells that play an essential role in tissue homeostasis, host defense, and the pathogenesis of many diseases, including cancer, cardiovascular and neurological disorders [1–3]. Macrophages can adopt functions ranging from pro-inflammatory to anti-inflammatory phenotypes [4–6]. However, the status and function of macrophages in the tumor microenvironment (TME) are highly plastic and heterogeneous [7]. Given this complexity, imaging total macrophage burden in the TME may be valuable for determining prognosis, monitoring treatment response, and understanding resistance mechanisms of immunotherapies in solid tumors like breast cancer [8, 9].

Histopathological examination of biopsy specimens remains the gold standard for macrophage detection, yet it is invasive and limited to small, localized tissue areas. Noninvasive imaging modalities such as positron emission tomography (PET) or single-photon emission computed tomography (SPECT) offer quantitative and sensitive approaches for macrophage detection in disease progression and treatment response. Several imaging probes have been developed for different macrophage markers. For example, [^64^Cu]Cu-ICT-01 targets CD163-positive M2-like macrophages and shows potential as a biomarker for vulnerable atherosclerotic plaques, while [^68^Ga]Ga-NOTA-anti-CD206 single-domain antibody and FDA-approved [^99m^Tc]Tc-Tilmanocept both target CD206 expressed on M2-like macrophages and dendritic cells [10–12]. Similarly, [^68^Ga]Ga-NOTA-MSA has been investigated for mannose receptor-positive M2-like macrophages in myocarditis [13]. Another avenue of imaging probes has been to target translocator protein (TSPO) with a large focus on neuroinflammation, but TSPO is not exclusive to macrophages and genetic polymorphisms in different individuals impact the binding affinity of these probes [14, 15]. Finally, ^64^Cu-CD68-Fc is a mouse fusion protein that leverages the Cluster of Differentiation CD68 (CD68) antigen as the probe to target oxidized LDL, thereby indirectly targeting macrophages and other scavenger receptors like dendritic and endothelial cells [16]. Despite these advances, most available tracers preferentially target the immunosuppressive subset of macrophages, exhibit poor specificity for macrophages, or indirectly targets macrophages, limiting their ability to accurately detect total macrophage burden in inflammatory diseases.

Given the limitations of existing macrophage imaging biomarkers, we aimed to directly target CD68, the gold-standard histopathological biomarker for pan-macrophage [17, 18]. CD68 is enriched in macrophages as both a cell surface and intracellular marker with lower total expression observed in other cell types [19]. Recently, our group demonstrated the feasibility of imaging cell surface CD68 on mouse macrophages in the TME of a syngeneic tumor model of renal cell carcinoma [20]. In this animal model with an intact immune system, we demonstrated that a radiolabeled anti-mouse CD68 antibody binds selectively to mouse CD68-positive macrophages over tumor cells in vitro and high uptake in tumor-associated macrophages (TAMs) and macrophage-rich organs in vivo. Therefore, we reasoned that combining the high affinity and specificity of antibodies with the sensitivity of PET imaging would allow direct targeting of CD68-positive human macrophages. To this end, we sought to establish the feasibility of targeting CD68 on the cell surface of macrophages by employing a phage displayed-Fab screening approach to discover novel Fabs specific to human CD68 and develop a Fab-based immunoPET tracer. This study describes the discovery of these Fabs and development of the lead Fab as an immunoPET tracer for human macrophages. Herein, we evaluate the feasibility of targeting cell surface CD68 in the TME.

## Materials and Methods

### Identification of Fabs specific to CD68 by phage display screening

**Fig. 1:**
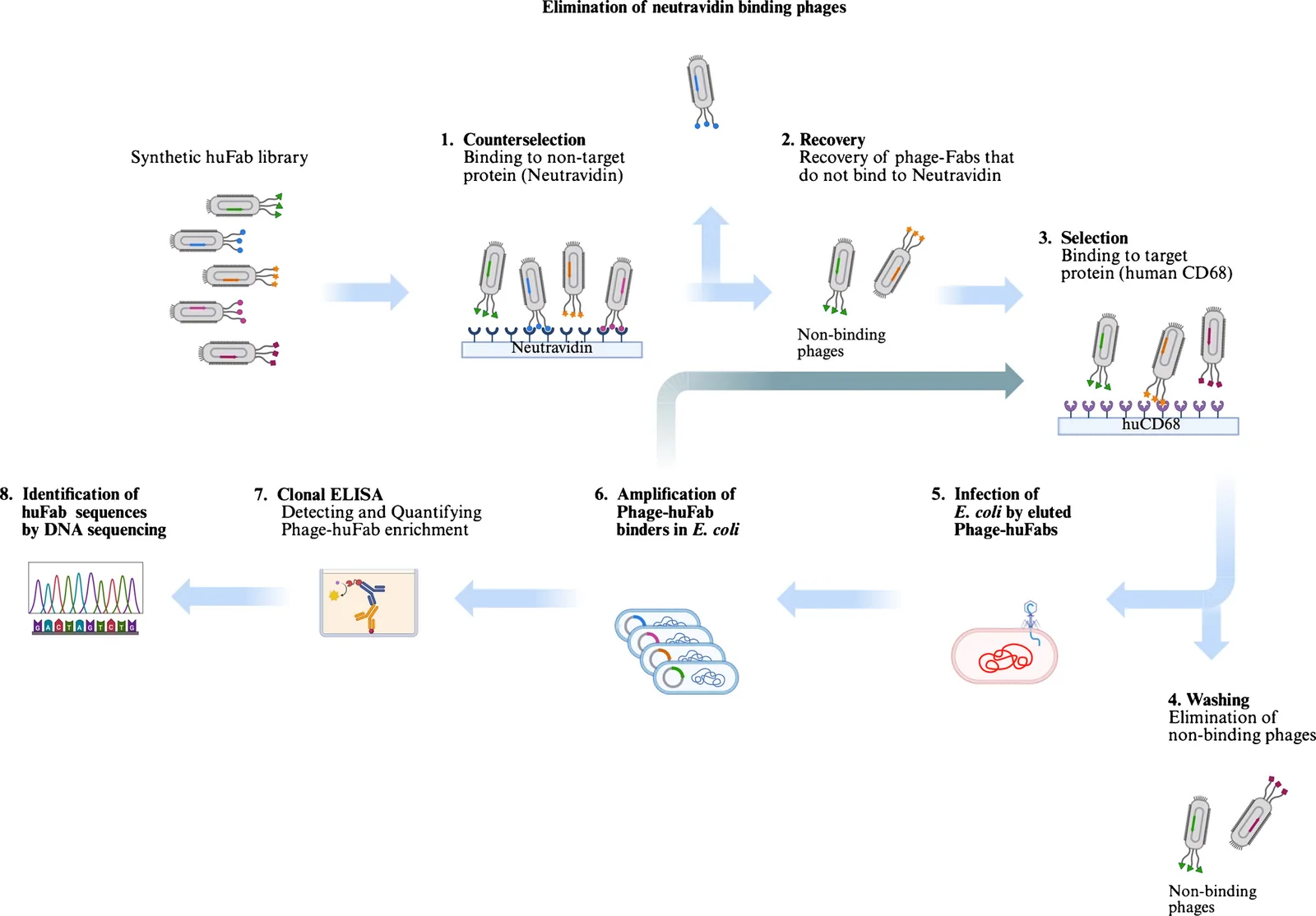
**Scheme of phage display screening of a synthetic Fab library.** Steps 3–6 were repeated 5 times, as indicated by the arrow from step 6 back to step 3. Created in BioRender. Marquez-Nostra, B. (2026) https://BioRender.com/d72x294

Phage Library F was previously designed without lysine residues in its complementarity determining regions (CDRs) [21]. Rather, lysines were replaced with arginine residues to maintain this basic amino acid property within the CDRs without a nucleophilic function. The framework contains several lysine residues to facilitate random conjugation with the amine-reactive chelator without interfering with the Fab's immunoreactivity. Library F was screened to identify binders against immobilized recombinant human CD68 (huCD68; Sino Biological; 11,192-H02H). The screening followed previously described methods (Fig. 1) [21, 22]. Specific details are described in Supplementary Material. Each unique sequence of Fab will be referred to as Fab followed by a number hereafter (e.g. Fab1, Fab2, Fab3, etc.).

### Expression, purification, and characterization of Fabs

The expression and purification of soluble Fabs (without phage) were conducted following previous methods [21]. *E. coli* cultures (150 mL) of Fab clones were allowed to grow until the optical density (OD) at 630 nm reached 0.8—1.0 followed by induction with 0.01 mM isopropyl-β-D-thiogalactopyranosid (IPTG; Sigma-Aldrich; I5502-5G) for about 3 h at 37 °C. Cultures were then centrifuged and the resulting bacterial pellets were stored at −80 °C overnight. Cells were lysed with 10 mL of lysis buffer (Table S1) for 1 h at 4 °C. Lysates were centrifuged and the supernatant containing Fabs were purified by Protein-A (Cytiva; 17,127,902) chromatography. The chemical identity of the Fabs was confirmed via SDS-PAGE (150 V, 40 min) using 4–20% stain free gel (BioRad; 4,568,093). Protein concentration was determined by BCA assay (ThermoFisher; 23,227).

### Characterization of Fab binding to huCD68

ELISA was performed on immobilized human or mouse CD68 (Sino Biological Inc.) to determine the binding cross-reactivity of the Fabs to the CD68 orthologs. Mouse or human CD68 (50 *μ L* of 0.5 $\mu \mathrm{g}/\mathrm{m}\mathrm{L}$) was immobilized in 96-well Maxisorp plates, overnight at 4 °C. ELISA was performed according to previous methods [22]. Absorbance was measured at OD_450nm_. We then determined the half-maximal effective concentration (EC_50_) of each huCD68-binding Fab by ELISA as described above. Different concentrations of the Fabs were added to wells containing huCD68 or BSA. Absorbance intensities were plotted against the log concentration of added Fab. A nonlinear regression analysis was applied, fitted using a dose–response curve to calculate EC_50_ values.

### Conjugation and radiolabeling

**Fig. 2:**
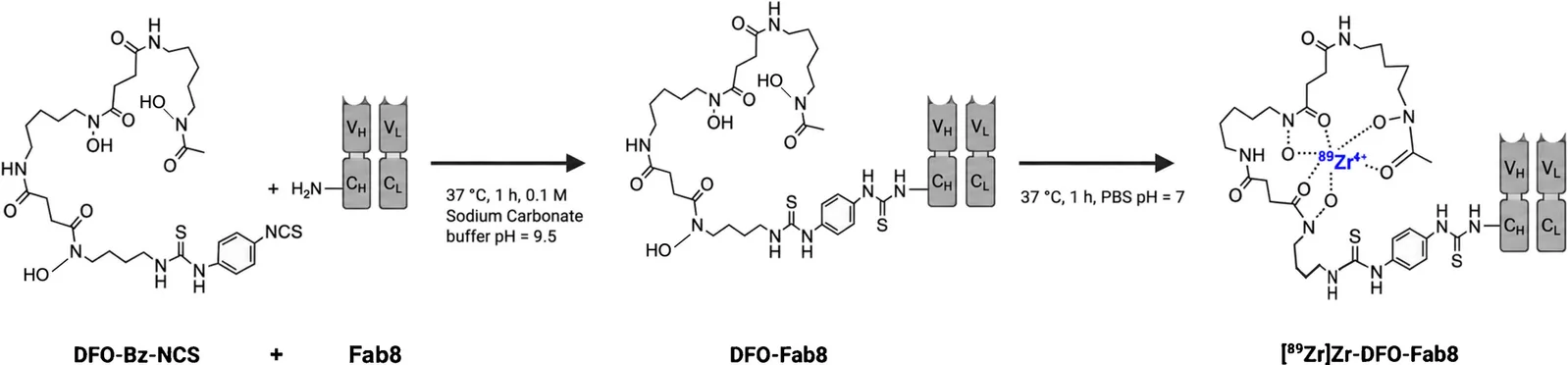
**Conjugation and radiolabeling scheme for the synthesis of [**^**89**^**Zr]Zr-DFO-Fab. **Created in BioRender. Vaughn Embs, A. (2026) https://BioRender.com/dm8p79h

The lead Fab was conjugated to the p-SCN-Bn-Deferoxamine (p-SCN-Bn-DFO) and radiolabeled with ^89^Zr to obtain [^89^Zr]Zr-DFO-Fab8 following recommended procedures [23]. [^89^Zr]Zr-oxalate was purchased from the UAB Cyclotron Facility. DFO-Fab conjugate and an irrelevant Fab control (AntibodiesOnline; ABIN964336) were each radiolabeled in PBS with neutralized ^89^Zr-oxalate at a specific activity of 7.4 kBq/nmol and incubated for 1 h at 37 °C. Conjugation and radiolabeling conditions were optimized, and the radioconjugates were characterized via radio-SEC HPLC. Details are described in the Supplementary Materials and the synthesis scheme is depicted in Fig. 2.

### Stability studies

We evaluated the stability of unlabeled or [^89^Zr]Zr-DFO-Fab8 in PBS or in human serum. Purity (% monomer) was quantified by radio-size exclusion chromatography HPLC (radio-SEC-HPLC). Fab8 and [^89^Zr]Zr-DFO-Fab8 in PBS were incubated at ambient temperature at different timepoints (0-, 2-, 4-, 6-, 24-, and 48-h post addition to PBS). Serum stability was evaluated by adding 19 MBq [^89^Zr]Zr-DFO-Fab8 in human serum and incubating at 37 °C. The percentage of monomer was calculated by determining the fraction of the monomer peak area relative to the total area under all peaks in the chromatogram.

### Cell culture and macrophage differentiation

THP-1 (ATCC; TIB-202), a human monocytic leukemia cell line, was cultured in RPMI media according to ATCC instructions without the addition of 2-mercaptoethanol. Cells were authenticated by ATCC before and at the conclusion of these studies using short tandem repeat (STR) profiling. Cells were used for experiments up to passage number 25. THP-1 cells were differentiated to macrophages following previous methods [24]. HuCD68 expression was verified by flow cytometry and staining details are described in the Supplementary Materials.

### Radioligand binding on THP-1 macrophages

A 24-well plate was seeded with 0.25 million cells and differentiated as described above. Cells were washed with PBS, followed by fixation in 200 µL 4% paraformaldehyde (ThermoFisher; AA473779M) for 10 min at 4 ºC. Wells were washed three times with 200 µL PBS for 5 min each time. Nonspecific sites were blocked with 1% FBS in PBS for 30 min, followed by washing with PBS. After washing, 74 kBq of either [^89^Zr]Zr-DFO-Fab8 (50 nM) or [^89^Zr]Zr-DFO-Irrelevant Fab (400 nM) was added to each well and incubated for 2 h at ambient temperature and then washed with 200 µL of PBS, three times. Cells were lysed and were assayed in an automatic gamma counter (2470 Wizard^2^ Perkin Elmer) according to our previous method [25].

### Viability study

THP-1 macrophages were plated in a tissue culture-treated 96-well plate at 50,000 cells per well (100 µL). Serial dilutions of Fab8 and Triton X-100 (ThermoFisher; AAA16046AE) starting at 130 nM and 0.3% respectively were added to the wells. The plate was then incubated for 24 h at 37 °C and 5% CO_2_ and CellTiter-Glo ® 2.0 Reagent (Promega; G9241) was added to the wells according to the supplier instructions.

### PET imaging in huCD68 plugs

All animal studies were conducted in accordance with an approved IACUC protocol. HuCD68 plugs were formulated by mixing Matrigel basement membrane with 10 µg of either huCD68 protein or BSA (nonspecific control protein) in a 5:1 volumetric ratio to form “plugs” to a total volume of 100 *μ L* per plug. HuCD68 plugs were implanted subcutaneously in the right shoulder and BSA plugs in the left shoulder of BALB/c mice (Charles River Labs, Female, 4 months old) 1 h before injection of tracer. Mice were then injected with 100 µL of 930 kBq (6.3 µg mass dose) of [^89^Zr]Zr-DFO-Fab8 (n = 3) at specific activity of 7.4 kBq/nmol via tail vein injection. Under 1.5% isoflurane anesthesia, static PET scans were acquired for 30 min followed by CT acquisition at 1- and 4- h p.i. (Sofie GNEXT PET/CT). Subsequently, mice were euthanized, and tissues were harvested, weighed, and assayed in a gamma counter to assess biodistribution. The percentage of injected dose per gram of organ (% ID/g) was calculated.

### PET imaging of intratumorally injected THP-1 macrophages in a TNBC xenograft model

MDA-MB-231 (ATCC; HTB26), a human triple negative breast cancer (TNBC) cell line that does not express CD68 [26], was cultured using IMDM supplemented with 10% FBS. NSG mice (Jackson Labs, female, 2 months old) were implanted with 100 µL of 5 million MDA-MB-231 cells (50 µL cells in PBS + 10 µL Matrigel basement membrane) subcutaneously to both the right and left shoulders (n = 3). Tumors were allowed to grow until they reached a size range of 100–300 mm^3^ as measured by calipers. The same mice were then intratumorally injected with 50 µL (40 µL of 5 million cells in PBS + 10 µL Matrigel basement membrane) of THP-1 undifferentiated cells into the left tumors and THP-1 macrophages into the contralateral right tumors at 1 h before tracer injection. Mice were then injected with 100 µL of 930 kBq (6.3 µg mass dose) [^89^Zr]Zr-DFO-Fab8 and PET imaging was performed at 1- and 6- h p.i. followed by a biodistribution study as described above.

### PET/CT analysis

Activity concentrations were decay-corrected to the time of injection to animals. Tracer uptake in plugs, tumors and normal organs was quantified by CT-guided segmentation of whole tissues and determination of mean standardized uptake values (SUV_mean_) using Imalytics Preclinical analysis software (Gremse-IT GmbH, Aachen, Germany). Voxel histogram analysis was performed to quantify the heterogeneity of tracer uptake in the plugs or tumors as previously described [27].

### Immunofluorescence of THP-1 macrophages in TNBC tumors

Staining of huCD68 was performed using rabbit anti-human-CD68 antibody (1:1000; Abcam [EPR20545]; AB213363) in a humid chamber at 4 ºC overnight. The anti-rabbit-Alexa Fluor 647 (1:1000; Abcam; AB150063) secondary antibody was incubated at ambient temperature for 1 h. DAPI (NucBlue™ Fixed Cell ReadyProbes™ Reagent; ThermoFisher; R37606) was used to stain the nuclei at ambient temperature for 30 min. Two to three non-overlapping areas in the tumor core of each slide were imaged at 20 × magnification using confocal microscopy (Carl Zeiss Microscopy GmbH, Oberkochen, Germany). The number of CD68 positive cells (pink) per area was quantified using ImageJ software.

### Statistical evaluation

Data were analyzed using GraphPad Prism version 10.4.0 (GraphPad Software Inc., La Jolla, CA) and are presented as mean ± standard deviation (SD). All group numbers are n = 3 and a p-value of < 0.05 was considered statistically significant.

## Results

### Identification and characterization of CD68-binding Fabs

Phage display selection yielded 14 unique sequences. However, only five of these sequences were successfully subcloned into the expression vector — Fab3, Fab4, Fab7, Fab8 and Fab14. The five Fabs yielded 0.5–2 mg of Fab from 150 mL cultures at concentrations ranging from 0.4–1.4 mg/mL in 1.3 mL after Protein A purification.

**Fig. 3:**
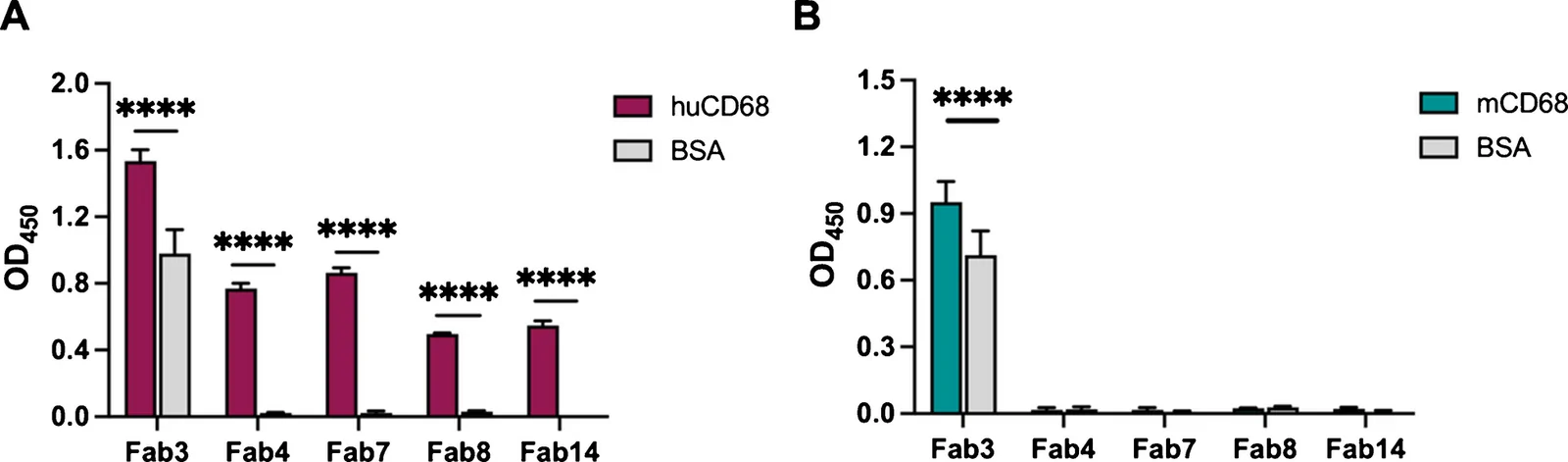
**Evaluation of binding specificity and cross-reactivity of lead Fabs.A.** ELISA of Fabs to huCD68 compared to BSA. **B.** ELISA of Fabs to mouse CD68 compared to BSA. ****p < 0.0001

We then characterized the specific binding of each Fab to huCD68 using a standard ELISA, where huCD68 or mouse CD68 (mCD68) were immobilized. Fab3, Fab4, Fab7, Fab8 and Fab14 bound to huCD68 significantly (p < 0.0001) compared to the BSA negative control using two-way ANOVA analysis (Fig. 3A). Although greater binding of Fab3 to huCD68 than to BSA was observed (p < 0.0001), its binding to BSA was considered high (OD_450_ = 0.98 ± 0.14). In contrast, the remaining Fabs (4, 7, 8, and 14) had minimal binding to BSA with OD_450_ values of less than 0.05. Furthermore, we evaluated each Fab's potential cross-reactivity to mCD68 and found that none of the Fabs except for Fab3 bound to mCD68 (Fig. 3B). However, Fab3 also bound nonspecifically to BSA (Fig. 3A-B). Thus, we eliminated Fab3 as a lead candidate.

**Fig. 4:**
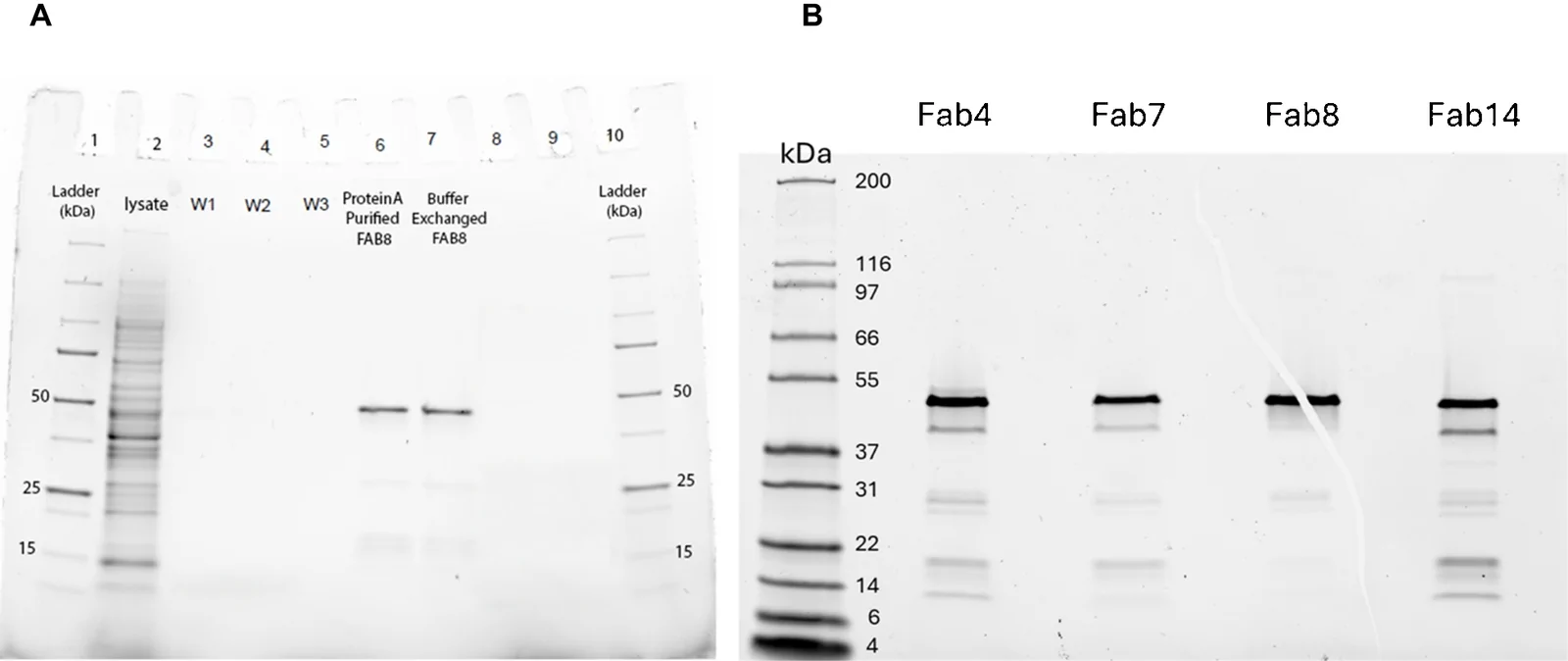
**SDS‑PAGE analysis of Fabs under nonreducing conditions. A.** Example of Protein A purification and buffer exchange results for Fab8.** B**. Comparison of the purity of Fab4, Fab7, Fab8, Fab14 after storage in 4 °C for 60 days

Based on ELISA data, we then characterized the purity of the lead Fabs. Purities were determined to be the following: Fab4 (99%), Fab7 (95%), Fab8 (99%), and Fab14 (96%) based on percent monomer by SEC-HPLC (Fig. S1). An example of SDS-PAGE analysis from each step of Fab8 purification is shown in Fig. 4A and confirms the molecular weight of the expressed Fab to be ~ 50 kDa. This analysis was also used to determine the purity and storage stability in the elution buffer of each Fab at 4 °C for 60 days, yielding the following monomeric purities: Fab4 (71%), Fab7 (75%), Fab8 (96%), and Fab14 (50%), based on ImageJ quantification of pixel intensities of the 50 kDa band relative to the total pixel intensities of all bands in the same lane (Fig. 4B).

**Fig. 5:**
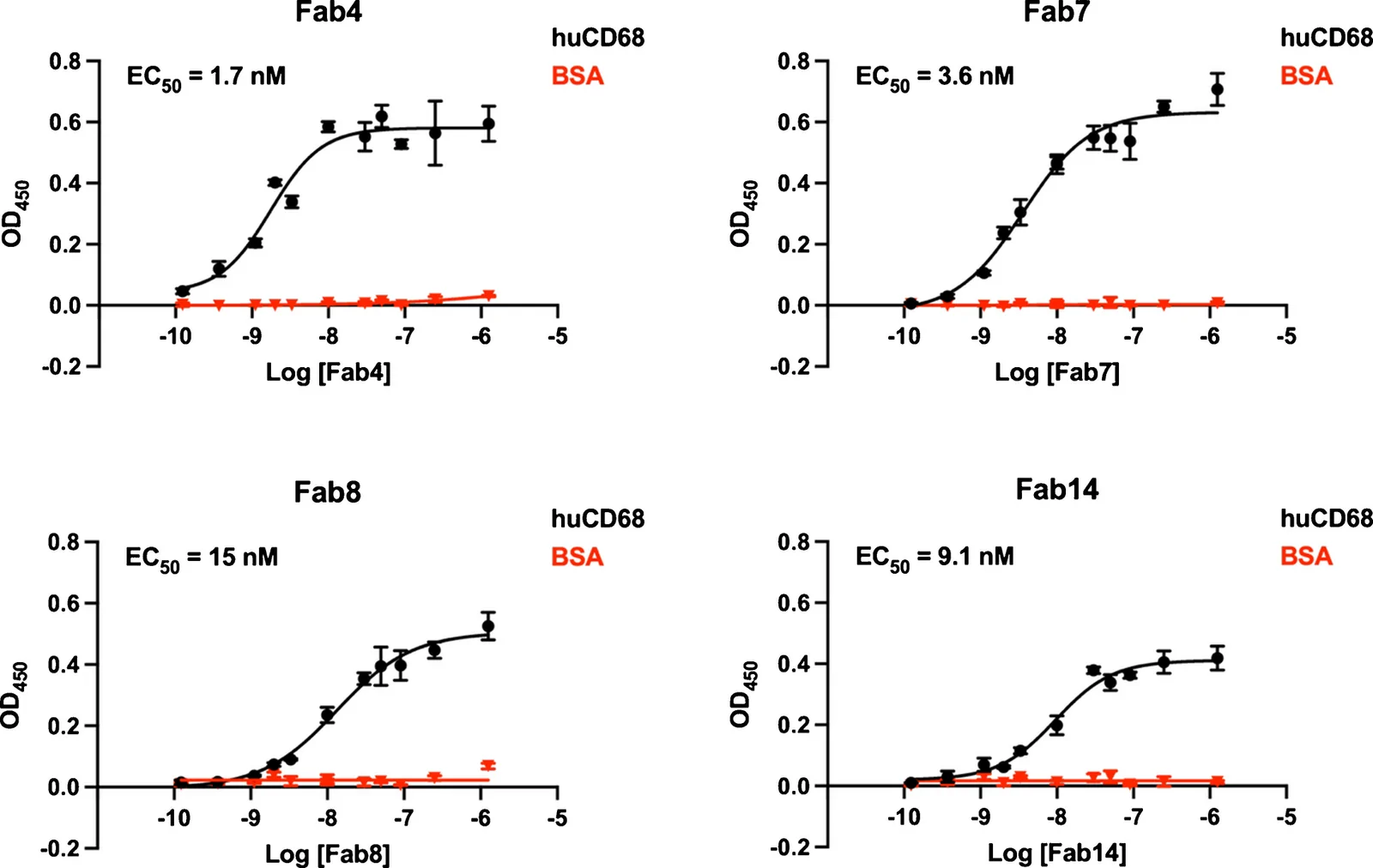
**Apparent binding affinities of lead Fabs for huCD68**

We then determined whether the Fabs’ binding to huCD68 is concentration-dependent to gain an understanding of their apparent binding affinities through EC_50_ values. Fab4, Fab7, Fab8, and Fab14 all bound specifically to huCD68 in a concentration-dependent manner, displaying the classic sigmoidal curve with EC_50_ values ranging from 1.7–15 nM (Fig. 5). Again, no binding to BSA was observed at all added concentrations. Nonlinear regression analysis was used to fit the dose–response curves. This strong apparent binding affinity in the nanomolar range is an important criterion for good immunoPET imaging probes.

### Identification of the lead Fab

To identify our lead Fab to move forward for immunoPET imaging applications, we evaluated the following metrics: specific binding, consistency in production, expression yields, and purity. Fab4, Fab7, Fab8, and Fab14 all had nanomolar EC_50_ values, which are suitable for immunoPET imaging applications. Fab production metrics became the deciding factor. We set an average of 1.7 mg/L expression yields, as this concentration is acceptable for the next stage of probe development – chemical conjugation and radiolabeling. Despite their lowest EC_50_ values, the expression yields of Fab4 (1.5 ± 0.81 mg/L) and Fab7 (1.2 ± 0.67 mg/L) did not meet this threshold consistently. Furthermore, they were unstable in storage conditions with purities of 71% and 75%, respectively (Fig. 3B). Fab14 reliably expressed in high yields (2.9 ± 0.76 mg/L) but with lower purity of 60–70%. Although the highest EC_50_ value for huCD68, Fab8 consistently produced good yields (1.7 ± 0.66 mg/L) along with the highest purity (80—90%) from protein A purification alone. Taken together, we chose Fab8 as our lead probe for development into an immunoPET probe. The remaining Fabs, which also demonstrated excellent binding specificity to huCD68 antigen, will require more optimization in production and storage conditions to give similar or better stability than that of Fab8.

### Conjugation and radiolabeling with Zirconium-89

To make an immunoPET probe, a chelator must be conjugated to the Fab under conditions which do not form protein aggregates followed by radiolabeling at the highest specific activity possible. We chose deferoxamine (DFO) as the chelator and ^89^Zr as the radioisotope due to their well-established chemistry and history of clinical translation. The conjugation condition with a stochiometric ratio of 4:1 (DFO:Fab) was selected since this condition yielded the least amount of unconjugated chelator without increased protein aggregation (Fig. S2). We then varied specific activities from 7.4 to 19 kBq/nmol to achieve the highest radiochemical yield as determined by radio-iTLC at ≥ 95%. The radio-iTLC chromatogram showed that a specific activity of 7.4 kBq/nmol of [^89^Zr]Zr-DFO-Fab8 achieved a radiochemical yield of 99% (Fig. S3). Therefore, [^89^Zr]Zr-DFO-Fab8 was produced at a specific activity of 7.4 kBq/nmol for all subsequent studies in vitro and in vivo.

### Stability studies

**Fig. 6:**
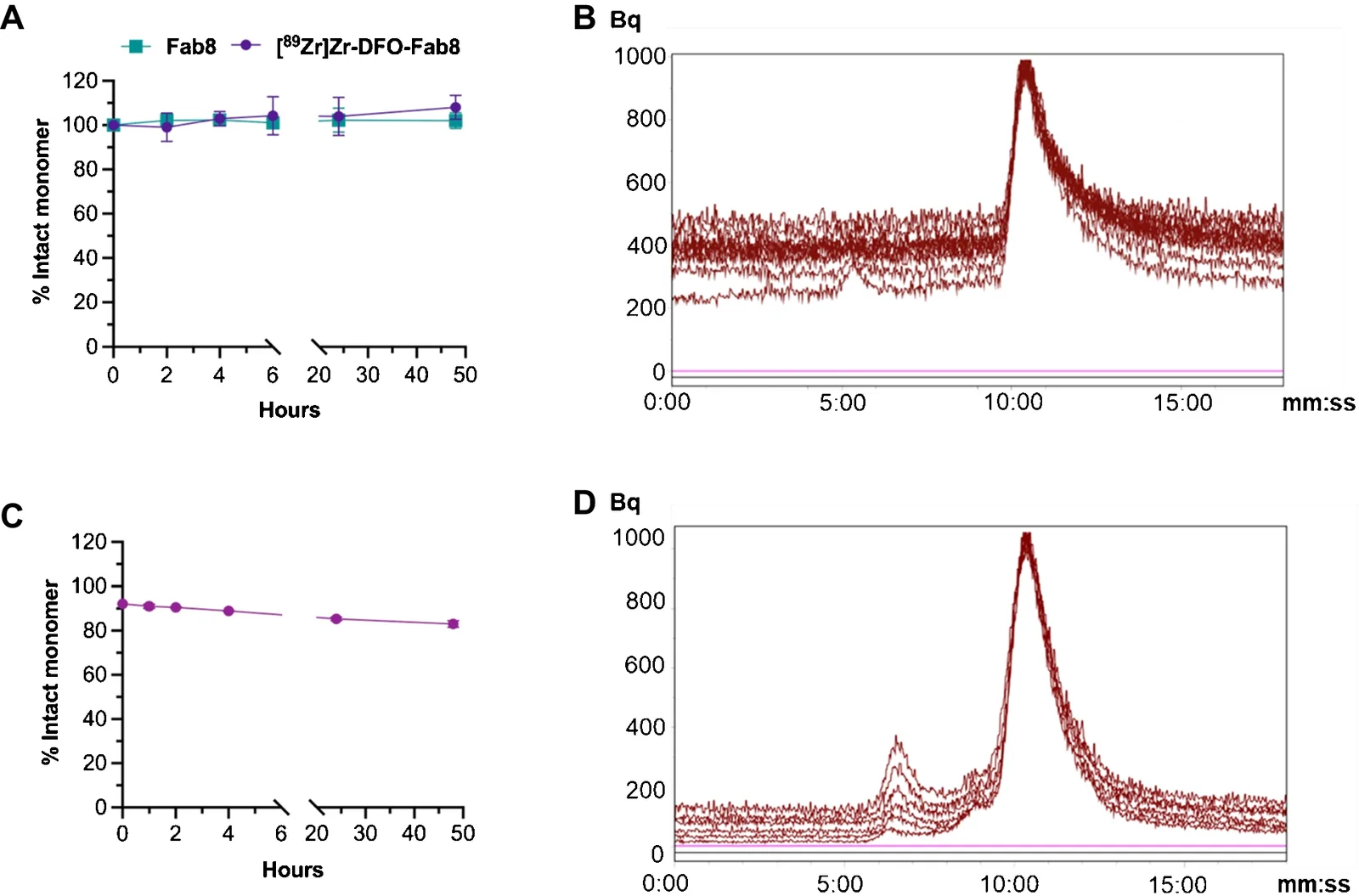
**Stability of Fabs in varying conditions over 48 h.A.** Stability of Fab8 and [^89^Zr]Zr-DFO-Fab8 in PBS. **B.** Radio-SEC-HPLC overlays of [^89^Zr]Zr-DFO-Fab8 in PBS. **C.** Stability of [^89^Zr]Zr-DFO-Fab8 in human serum. **D.** Radio-SEC-HPLC overlays of [^89^Zr]Zr-DFO-Fab8 stability in human serum

We evaluated the stability of both unlabeled Fab8 and [^89^Zr]Zr-DFO-Fab8 in PBS over 48 h by radio-SEC-HPLC and the intact monomer percentage remained stable at > 95% at a retention time of 11 min (Fig. 6A-B). Since Fab8 is specific for huCD68 we next evaluated its stability in human serum. [^89^Zr]Zr-DFO-Fab8 exhibited a slight decrease in intact monomer over time, but 80% remained as a monomer at a retention time 11 min after 48 h (Fig. 6C-D). The remaining 20% of radioactivity was associated with serum proteins at a retention time of 6.5 min. The retention times remained consistent over the 48-h study (Fig. 6B, D).

### Radioligand binding and viability of THP-1 macrophages

THP-1 macrophages are known to induce greater huCD68 expression compared to THP-1 undifferentiated cells [28]. These cells served as our biological model to assess the binding specificity of [^89^Zr]Zr-DFO-Fab8. The suspension cell line THP-1 cells change morphology to THP-1 macrophages, which become strongly adherent to plastic surfaces after treatment with PMA (Fig. S4). Flow cytometry analysis of both cell morphologies revealed 25 ± 0.61% huCD68 expression in THP-1 macrophages and 4.3 ± 0.36% in THP-1 undifferentiated cells (p < 0.0001 based on an Unpaired t test with Welch’s correction; Fig. S5-6). Cellular granularity of THP-1 macrophages was determined to be greater than THP-1 undifferentiated based on side scatter analysis; thus further validating its differentiation into macrophages (Fig. S5).

**Fig. 7:**
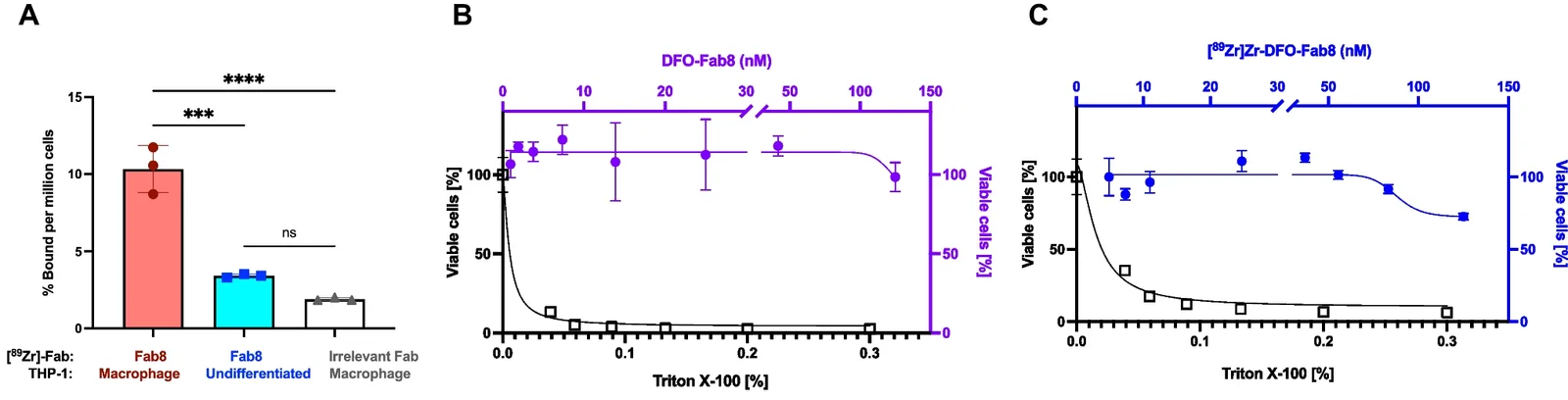
**Radioligand binding assay and effect of [**^**89**^**Zr]Zr-DFO-Fab8 on the viability of THP-1 macrophages. A**. Radioligand binding of [^89^Zr]Zr-DFO-Fab8 is greater in THP-1 macrophages than in undifferentiated cells. [^89^Zr]Zr-DFO-Fab8 shows binding specificity to CD68-positive THP-1 macrophages vs. [^89^Zr]Zr-DFO-Irrelevant Fab. ***p < 0.001, ****p < 0.0001 **B.** Effect of DFO-Fab8 and **C.** [^89^Zr]Zr-DFO-Fab8 on the viability of THP-1 macrophages

After confirming huCD68 expression in THP-1 macrophages, we evaluated the binding specificity of [^89^Zr]Zr-DFO-Fab8 using an irrelevant Fab tracer, [^89^Zr]Zr-DFO-Irrelevant Fab, as a negative control. [^89^Zr]Zr-DFO-Fab8 had significantly higher with 10 ± 1.5% binding than [^89^Zr]Zr-DFO-Irrelevant Fab with 2.0 ± 0.097% binding to THP-1 macrophages (p = 0.0002 based on a one-way ANOVA with multiple comparisons; Fig. 7A). Additionally, binding of [^89^Zr]Zr-DFO-Fab8 was significantly greater in THP-1 macrophages than in THP-1 undifferentiated cells with only 3.4 ± 0.11% binding (p < 0.0001; Fig. 7A).

Given the novelty of Fab8, we then evaluated the biological effect of DFO-Fab8 conjugate and radiolabeled conjugate on the viability of THP-1 macrophages, using Triton X-100 as a cell lysing control. Serial dilutions of Triton X-100 control showed a concentration dependent reduction in THP-1 macrophage viability, whereas the DFO-Fab8 or [^89^Zr]Zr-DFO-Fab8 did not have a detrimental effect on THP-1 macrophage viability at the concentrations tested; a small decrease in viability was found at high [^89^Zr]Zr-DFO-Fab8 concentration at 125 nM where the viability decreased by 19% (Fig. 7B-C). The concentration range was chosen based on estimated tracer concentrations used for animal studies in vivo (1—5 nM), estimating a 2-mL blood volume for a mouse.

### PET imaging of huCD68 antigen plugs

**Fig. 8:**
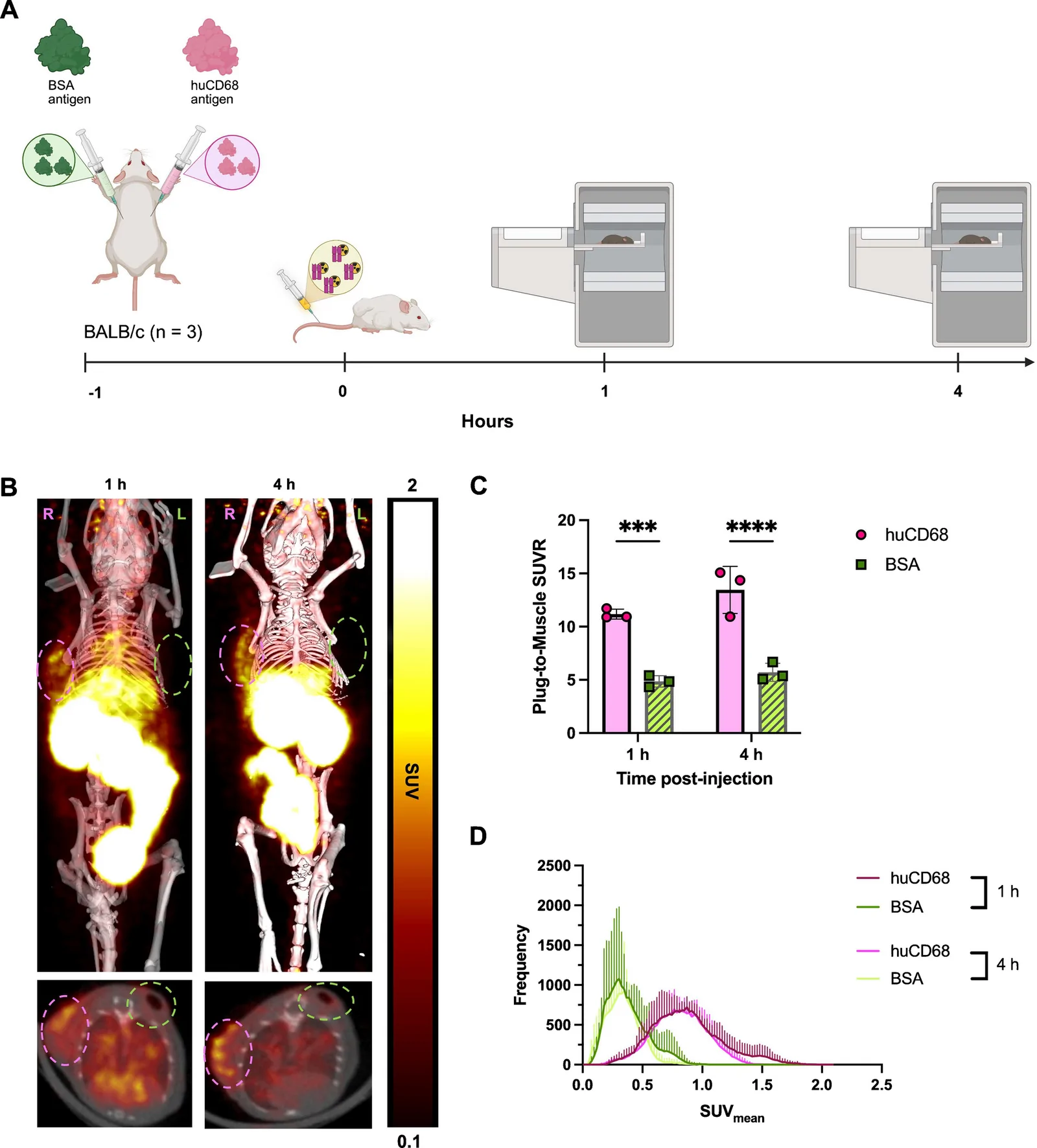
**PET/CT imaging of huCD68 and BSA plugs. A.** Scheme of experimental design**. B.** Representative PET/CT MIP and axial view. **C.** Plug-to-Muscle SUVR at 1- and 4- h p.i. represented as Standardized Uptake Value Ratio (SUVR). **D.** Voxel intensity histograms of the frequencies of the SUV_mean_ in the antigen plug model at 1- and 4- h p.i. R – right plug (huCD68 antigen) in pink L – left plug (BSA antigen) in green. Dashed circles mark antigen plug locations. ***p = 0.0002 ****p < 0.0001. Created in BioRender. Vaughn Embs, A. (2026) https://BioRender.com/iyimt94

Fab8 is highly specific to huCD68 antigen with no cross-reactivity to mCD68. Therefore, the huCD68 antigen plug model (Fig. 8A), to which Fab8 demonstrated specific binding in vitro, was the most suitable initial animal model for evaluating the pharmacokinetic properties and specificity of [^89^Zr]Zr-DFO-Fab8 in vivo. Figure 8B shows the maximum intensity projections (MIP) and the axial views. PET/CT imaging of [^89^Zr]Zr-DFO-Fab8 demonstrated specific uptake in the huCD68-plugs with minimal uptake in the contralateral BSA-plugs at 1- and 4-h p.i. as determined by the SUV_mean_ (p = 0.0006, p = 0.0008, respectively; Fig. S7A). Next, we determined the SUV ratios (SUVR) of Plug-to-Heart (PHR), Plug-to-Muscle (PMR) and huCD68-to-BSA. The PHR of [^89^Zr]Zr-DFO-Fab8 in the huCD68-plugs was determined to be 0.99 ± 0.20 and 2.2 ± 0.014 at 1- and 4-h p.i., respectively (Fig. S7B). In contrast, the PHR in the BSA-plugs were 0.43 ± 0.10 and 0.92 ± 0.14 at 1- and 4-h p.i., respectively. The PHR were significantly higher than the BSA-plugs at 1- and 4-h p.i. (p = 0.0099, p < 0.0001 respectively; Fig. S7B**)**. The PMR for the huCD68-plugs was determined to be 11 ± 0.46 and 13 ± 2.2 at 1- and 4-h p.i., respectively (Fig. 8C). In contrast, the PMR in the BSA-plugs were 4.9 ± 0.53 and 5.7 ± 0.85 at 1- and 4-h p.i., respectively. The PMR for the huCD68-plugs was significantly higher than that of the BSA-plugs at 1- and 4-h p.i. (p = 0.0002, p < 0.0001 respectively; Fig. 8C**)**. We then quantified the huCD68-to-BSA SUVR and observed no significant differences between 1- and 4-h p.i., demonstrating specific retention of the [^89^Zr]Zr-DFO-Fab8 (Fig. S7C).

To determine the distribution of tracer uptake within the plugs, voxel intensity histogram analysis of plug segmentations was used. These histograms demonstrated that the tracer distributions were consistent between both imaging timepoints for huCD68- and for BSA-plugs, albeit with lower overall intensity in the BSA plugs, as expected (Fig. 8D). We used the Kolmogorov–Smirnov (K-S) test to compare cumulative distributions. The K-S distance, which quantifies the maximum difference between two distributions, was greater for huCD68 plugs at 1 h (0.40 ± 0.12) and 4 h (0.30 ± 0.050) time points compared with BSA plugs, demonstrating specific uptake over time (p < 0.0001 and p = 0.0023, respectively). Table 1 shows the segmentations of normal organs demonstrating high uptake of kidneys, as to be expected with a tracer of this size. Biodistribution studies at 4 h p.i. in Table S2 demonstrate higher accumulation in kidneys (9-fold), liver (3-fold) and spleen (2-fold). In contrast, huCD68 plugs showed elevated uptake in comparison to BSA plugs (2-fold), lungs (2-fold), blood (3-fold), bone (4-fold), heart (3-fold), and muscle (11-fold). Additionally, the biodistribution highly correlated with SUV_mean_ values of segmented organs with a Pearson’s correlation coefficient (r) of 0.9702 (Fig. S7D).

**Table 1:** **Image derived biodistribution of [**^**89**^**Zr]Zr-DFO-Fab8 in BALB/c mice (n = 3) with bilateral antigen plugs**

**Tissue**	**SUV**_**mean**_** ± SD**	
	**1 h p.i**	**4 h p.i**
Lungs	0.90 ± 0.23	0.57 ± 0.072
Liver	1.6 ± 0.37	1.3 ± 0.15
Kidneys	8.2 ± 1.9	11 ± 1.7
Heart	0.90 ± 0.24	0.37 ± 0.015
huCD68 plug	0.87 ± 0.12	0.81 ± 0.056
BSA plug	0.38 ± 0.10	0.34 ± 0.046
Muscle	0.078 ± 0.013	0.061 ± 0.0078

### PET imaging of THP-1 macrophages in TNBC tumors

**Fig. 9:**
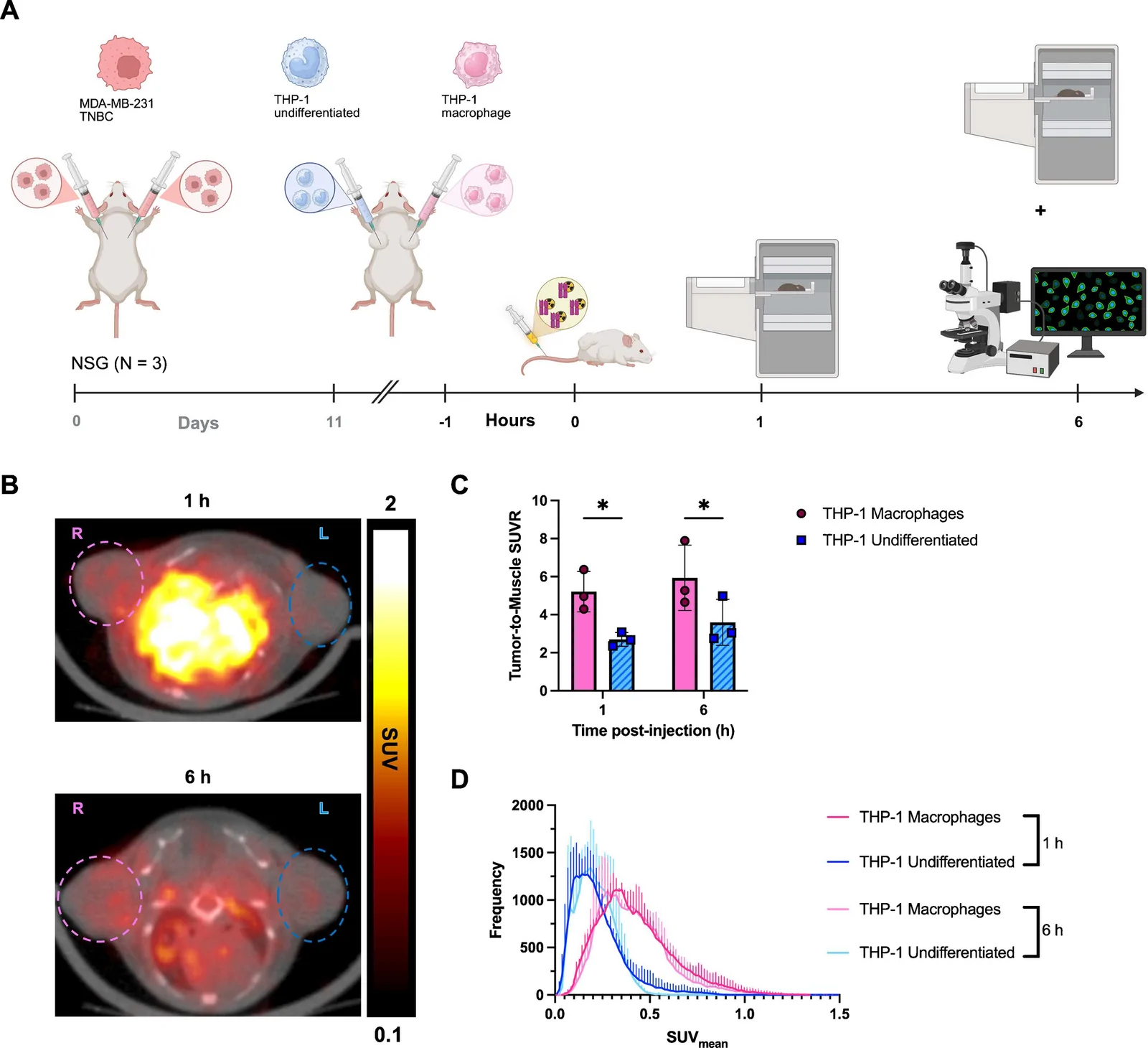
**PET/CT imaging of MDA-MB-231 tumors intratumorally injected with THP-1 macrophages. A**. Scheme of experimental design. **B.** Representative PET/CT axial view. **C.** Tumor-to-Muscle SUVR at 1- and 6-h p.i. **D**. Voxel intensity histograms of the frequencies of the SUV voxels in the tumor models at 1- and 6-h p.i.. R – right tumor (THP-1 Macrophage) in pink L – left tumor (THP-1 undifferentiated) in blue. Dashed circles mark tumor locations. *p = 0.0326 and *p = 0.0426. Created in BioRender. Vaughn Embs, A. (2026) https://BioRender.com/zud7bcm

The promising results of the antigen plug imaging led us to evaluate a more biologically relevant model, and that which contains the tumor vasculature to mimic the tumor microenvironment infiltrated with human macrophages. We developed a breast cancer xenograft model with bilateral MDA-MB-231 tumors in which THP-1 macrophages were intratumorally injected on the right side and THP-1 undifferentiated cells were injected in the contralateral tumor prior to administering the tracer (Fig. 9A). Figure 9B shows the axial views of PET/CT imaging at 1- and 6-h p.i.. PET/CT images demonstrated specific uptake at 1- and 6-h p.i. in THP-1 macrophage tumors vs THP-1 undifferentiated tumors as determined by SUV_mean_ (p = 0.0007 and p = 0.0064, respectively; Fig. S8A). Next, we determined the SUV ratios for Tumor-to-Heart (THR), Tumor-to-Muscle (TMR) and THP-1 macrophage-to-THP-1 undifferentiated. The THR of [^89^Zr]Zr-DFO-Fab8 in THP-1 macrophages was determined to be 0.34 ± 0.051 and 0.48 ± 0.069 at 1- and 6-h p.i. respectively. In contrast, the THR in THP-1 undifferentiated was 0.18 ± 0.027 and 0.29 ± 0.026, respectively (Fig. S8B). THP-1 macrophage tumors were significantly higher than the THP-1 undifferentiated tumors at 1- and 6-h p.i. (p = 0.0120, p = 0.0041 respectively; Fig. S8B). The TMR of [^89^Zr]Zr-DFO-Fab8 in THP-1 macrophages was determined to be 5.2 ± 1.1 and 5.9 ± 1.7 at 1- and 6-h p.i. respectively. In contrast, the TMR in THP-1 undifferentiated was 2.7 ± 0.36 and 3.6 ± 1.2, respectively (Fig. 9C). Uptake in the THP-1 macrophage tumors were significantly higher than in the THP-1 undifferentiated tumors at 1- and 6-h p.i. (p = 0.0326, p = 0.0426 respectively; Fig. 9C). Moreover, there was no significant difference in THP-1 macrophage-to-THP-1 undifferentiated SUVR between 1- and 6-h p.i., also demonstrating specific retention (Fig. S9C).

Voxel intensity histogram analysis demonstrated a broad, but significant shift towards higher SUV for the tracer uptake in tumors with THP-1 macrophages over those with THP-1 undifferentiated. K-S distances were calculated to be 0.37 ± 0.23 (p = 0.0004) and 0.27 ± 0.15 (p < 0.0001) at 1- and 6-h p.i., respectively (Fig. 9D). Table 2 shows the segmentations of normal organs demonstrating high uptake of kidneys, as to be expected with a tracer of this size. Biodistribution studies at 6 h p.i. in Table S3 demonstrate that the tracer had higher accumulation in normal organs like kidneys (44-fold), liver (12-fold), lungs (3-fold), and spleen (8-fold) relative to its uptake in the THP-1 macrophage-injected tumor. In contrast, tracer uptake in THP-1 macrophage-injected tumors was 3-fold higher than in THP-1 undifferentiated tumors and 9-fold higher than in muscle. Additionally, the biodistribution highly correlated with SUV_mean_ values of segmented organs with a Pearson’s correlation coefficient (r) of 0.9732 (Fig. S8D).

**Table 2:** **Image -derived biodistribution of [**^**89**^**Zr]Zr-DFO-Fab8 in NSG mice (n = 3) with bilateral MDA-MB-231 tumors with intratumoral THP-1 macrophages or THP-1 undifferentiated cells**

**Tissue**	**SUV**_**mean**_** ± SD**	
	1 h p.i	6 h p.i
Lungs	1.9 ± 0.25	1.8 ± 0.11
Liver	3.6 ± 0.36	3.7 ± 0.33
Kidneys	8.2 ± 0.97	9.2 ± 0.67
Heart	1.3 ± 0.15	0.73 ± 0.089
THP-1 Macrophages	0.42 ± 0.030	0.35 ± 0.027
THP-1 Undifferentiated	0.22 ± 0.051	0.21 ± 0.031
Muscle	0.082 ± 0.014	0.062 ± 0.012

### Immunofluorescence of THP-1 macrophages in TNBC tumors

**Fig. 10:**
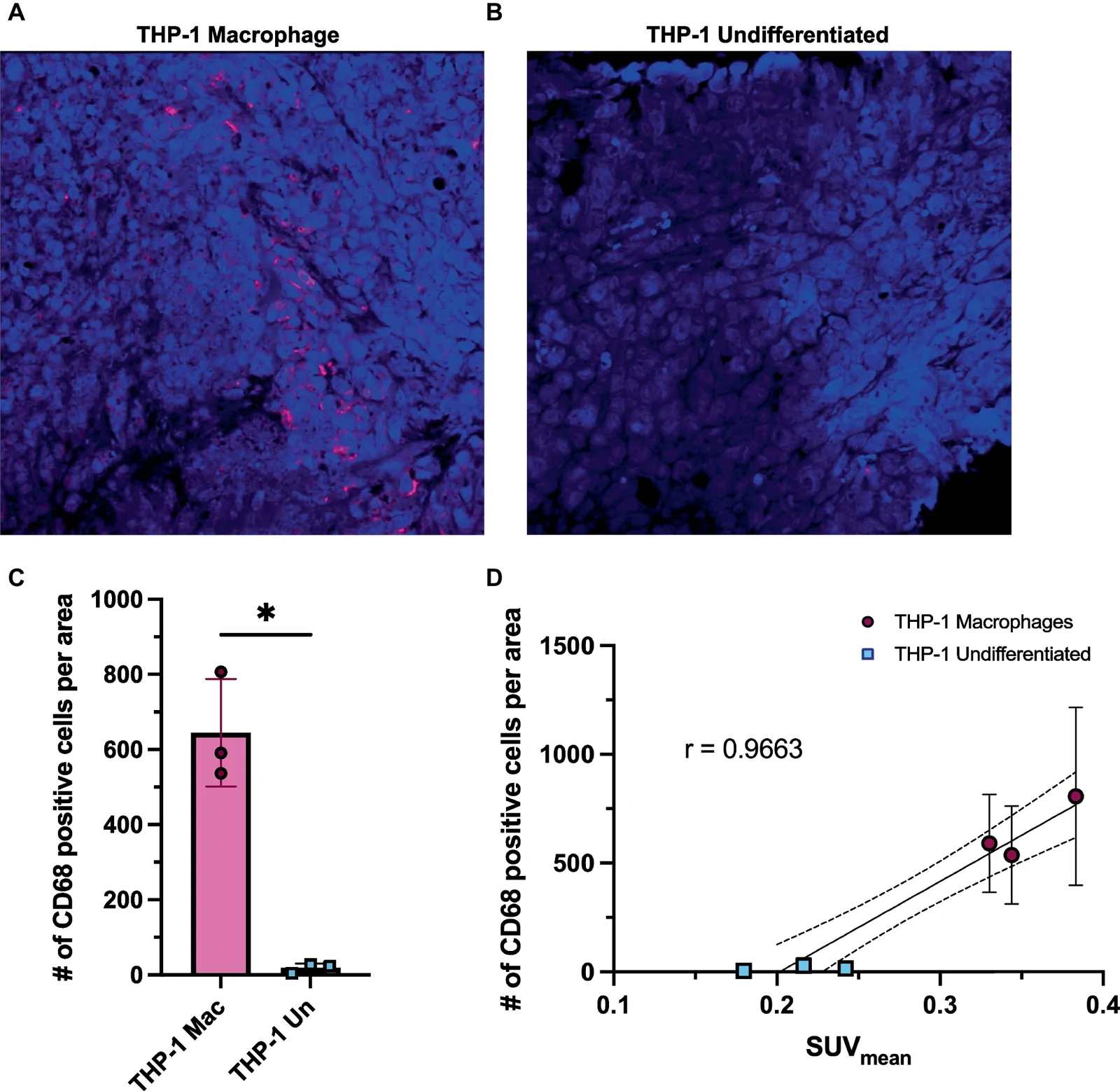
**IF staining of CD68 in MDA-MB-231 tumors intratumorally injected with Matrigel containing either THP-1 macrophages or undifferentiated cells.** Representative 20 × image of **A.** tumor with THP-1 macrophages and **B.** tumor with THP-1 undifferentiated cells. Blue, nuclei; pink, huCD68. **C.** Quantification of number of CD68 positive cells per area by IF. * p = 0.0164 **D.** Correlation between CD68 expression and PET SUV_mean_ of tumors (Pearson’s r = 0.9663). Simple linear analysis with 90% confidence interval was applied

To verify huCD68 expression biologically, we quantified surface huCD68 expression by IF in the TNBC tumors post PET imaging at 6 h. All H&E and IF images from individual tumors are presented in Fig. S9—S10. Figure 10A shows a 20 × IF image of THP-1 macrophage injected tumor, containing 660 ± 290 cells expressing huCD68. Figure 10B shows the THP-1 undifferentiated injected tumor, containing a significantly lower huCD68 expression with 16 ± 23 huCD68-positive cells (p = 0.0164 based on an Unpaired t test with Welch’s correction, Fig. 10C). These results showed a significantly high correlation with SUV_mean_ in the tumor with a Pearson’s correlation coefficient (r) of 0.9663 (Fig. 10D).

## Discussion

In cancer, several therapies targeting macrophages are currently under clinical and preclinical investigations [29]. Macrophage infiltration is associated with poor overall survival, a greater risk of metastasis and resistance to immunotherapy [30]. In breast cancer, high CD68-positive macrophage density is linked to aggressive features including larger tumor size, higher histologic grade, hormone-receptor negativity, lymphovascular invasion, decreased overall survival and reduced progression free survival [31, 32]. CD68 expression is also reported in some tumor cell lines and other non-myeloid cells, although positive expression is dependent on the antibody clone used for analysis [19]. We have previously shown that cell surface CD68 increases over time as monocytes become differentiated into macrophages [20]. Furthermore, the abundance of CD68-positive TAMs in the TME of a syngeneic tumor model permits selective imaging with an anti-mouse CD68 probe, establishing cell surface CD68 as a viable marker of TAMs [20]. Hence, an immunoPET probe that directly targets macrophages by binding to cell surface human CD68, could help with patient selection for macrophage modulating treatments, monitoring response to treatments, or predicting resistance to immunotherapy. In this work, we developed the first generation immunoPET probe that specifically targets huCD68 in vitro and in vivo. [^89^Zr]Zr-DFO-Fab8 is stable in vitro (Fig. 6) and binds specifically to CD68 + THP-1 macrophages without detrimentally affecting their viability in vitro (Fig. 7).

The absence of [^89^Zr]Zr-DFO-Fab8’s cross-reactivity to mouse CD68 posed a significant challenge with the selection of a suitable mouse model for evaluating its specificity in vivo. Initially, we evaluated our tracer in bilateral antigen plugs of huCD68 antigen and BSA (control antigen) to evaluate its pharmacokinetic properties in the presence of the target antigen. We used this method as a proof of concept to verify specific binding to huCD68 in vivo, despite the absence of vasculature in the antigen plug at our imaging timepoints [33]. Although the mechanism of action of tracer delivery in antigen plugs in vivo is not yet established to the best of our knowledge, we hypothesized that this mechanism involves passive diffusion of the tracer into the subcutaneously implanted plugs. Furthermore, we established the plugs in immunocompetent mice to evaluate the tracer's performance in targeting huCD68 antigen in the presence of an intact immune system. [^89^Zr]Zr-DFO-Fab8 demonstrated greater uptake in the huCD68-plugs compared with the BSA-plugs (Fig. 8). The limiting factor of this model is the uncertainty of antigen stability in the Matrigel plug over time. This issue did not permit imaging at later timepoints that is typical of ^89^Zr-labeled antibodies to improve imaging contrast [34]. Imaging at later time points or the use of shorter lived isotopes may be explored in future studies. Nevertheless, [^89^Zr]Zr-DFO-Fab8 binds specifically to huCD68 in vivo within the first few hours of tracer injection and also shows feasibility of using antigen plugs as a carrier of target antigens as viable, convenient animal models for pilot pharmacokinetic studies for new tracers.

After confirming specific binding to antigen plugs, we aimed to evaluate this binding in human cell derived xenografted tumors in vivo. MDA-MB-231 is an established tumorigenic cell line that forms highly vascularized tumors [26]. Furthermore, MDA-MB-231 cells do not express CD68 [35]. Moreover, intratumoral injections of immune cells have previously been established to generate other mouse models [36, 37]. Inspired by these studies, we generated a novel animal model of human macrophages in xenografted tumors to demonstrate feasibility of immunoPET imaging of human macrophages in vivo. To accomplish this goal, we implanted bilateral tumors and allowed vasculature to develop prior to intratumoral injections of either THP-1 macrophages or THP-1 undifferentiated cells on either side. Subsequently, the tracer was injected systemically followed by same-day PET/CT imaging. The results showed a significant increase in tracer uptake in THP-1 macrophages compared with THP-1 undifferentiated cells (Fig. 9).

It is important to note that the THP-1 differentiated cells with about 20% huCD68 expression were injected intratumorally, representing a minor fraction of the total tumor region. The entire tumor was segmented to determine SUV_mean_, which was then used to obtain SUVR with the muscle as the reference tissue or heart to compare circulating tracer. Without the ability to delineate the margins between the THP-1 macrophage cells from the tumor cells on CT, SUV_mean_ values were expected to be low. While tumor-to-muscle SUVR were consistently high (Fig. 9C), tumor-to-heart SUVR was less than 1 (Fig. S8B), suggesting a suboptimal clearance from the bloodstream at early imaging timepoints, as expected for antibody fragments of this size and possibly due to some protein aggregation in vivo. Nevertheless, [^89^Zr]Zr-DFO-Fab8 sensitively detected THP-1 macrophages in the TME, and its uptake is significantly higher than that in the THP-1 undifferentiated cells in the contralateral tumor. Finally, IF staining of CD68 confirmed that the tumors injected with THP-1 macrophages had significantly higher huCD68 expression than the THP-1 undifferentiated tumors and highly correlated with PET SUV_mean_ (Fig. 10). Thus, we demonstrated direct PET-to-histopathological correlation for the gold standard biomarker of pan-macrophages.

[^89^Zr]Zr-DFO-Fab8 achieved specificity for huCD68 in vivo but there is the limitation of high kidney and lung uptake which is consistent with other [^89^Zr]Zr-Fab tracers [38–41]. In vitro stability studies in serum showed approximately 10% aggregation, which could contribute to increased liver and spleen uptake via reticuloendothelial system sequestration. [42, 43]. High background signal is more apparent in this animal model because the target uptake is low. Future work includes improving the pharmacokinetic properties by reducing kidney uptake through incorporating high molecular weight polyethylene glycol (PEG). This approach is clinically relevant, as demonstrated by the approved Certolizumab Pegol for the treatment of Crohn’s disease or rheumatoid arthritis [44].

In summary, we developed [^89^Zr]Zr-DFO-Fab8 from discovery to preclinical immunoPET applications in novel huCD68 mouse models. [^89^Zr]Zr-DFO-Fab8 specifically targets huCD68-positive macrophages with high specificity and sensitivity, thereby representing the first-generation human CD68-targeted immunoPET tracer and opens an area of research to improve its pharmacokinetic properties along with better animal models for human macrophages prior to clinical translation of CD68-immunoPET for whole-body quantification of macrophage burden and monitoring response to treatment.

## Supplementary Information

Below is the link to the electronic supplementary material.

Supplementary file1 (DOCX 7349 KB)

## Data Availability

Imaging data will be made publicly available at the Preclinical Image Dataset Repository or upon reasonable request from the corresponding author.

## Abbreviations

TME: Tumor microenvironment
PET: Positron emission tomography
SPECT: Single-photon emission computed tomography
TSPO: Translocator protein
CD68: Cluster of differentiation 68
TAMs: Tumor-associated macrophages
CDR: Complementarity determining regions
huCD68: Human CD68
OD: Optical density
IPTG: Isopropyl-β-D-thiogalactopyranosid
SDS-PAGE: Sodium dodecyl sulfate–polyacrylamide gel electrophoresis
BSA: Bovine serum albumin
PBST: Phosphate buffered saline with 0.05% tween-20
EC_50_: Half maximal effective concentration
DFO: p-SCN-Bn-Deferoxamine
Radio-SEC-HPLC: radio-size exclusion chromatography high-performance liquid chromatography
ATCC: American Type Culture Collection
STR: Short tandem repeat
CT: Computed tomography
p.i.: Post-injection
% ID/g: Injected dose per gram of organ
TNBC: Triple negative breast cancer
DAPI: 4',6-diamidino-2-phenylindole
SD: Standard deviation
mCD68: Mouse CD68
radio-iTLC: Radio-instant-thin layer chromatography
MIP: Maximum intensity projection
SUV: Standardized uptake value
SUVR: Standardized uptake value ratio
PHR: Plug-to-heart
PMR: Plug-to-muscle
THR: Tumor-to-heart
TMR: Tumor-to-muscle
K-S: Kolmogorov–Smirnov
IF: Immunofluorescence
PEG: Polyethylene glycol
