# Supplementary material for: A FABulous approach to image human macrophages
Source: Eur J Nucl Med Mol Imaging. 2026 Sep 28;53(13):7770–85. doi: 10.1007/s00259-026-08180-y (PMC13727820; doi:10.1007/s00259-026-08180-y)
Supplement: Supplementary file 1 [file 259_2026_8180_MOESM1_ESM.docx]

**SUPPLEMENTARY MATERIALS**

**Title: A FABulous approach to image human macrophages**

**Authors & Affiliations**: Borna Roohani,^1,2*^ Ayla Vaughn Embs,^1*^ Robin Kumar,^1^ Supum Lee,^3^ Samantha R. Katz,^3^ Chloe La Prairie,^4^ Dajah Nash,^1^ Mann Dangarwala,^1^ Harriet M. Kluger,^5^ Daryl Klein,^6^ Ping Zhang,^4^ Benjamin M Larimer,^1^ Bryce Nelson,^7^ and Bernadette Marquez-Nostra^1#^

^*^Authors contributed equally to this work

^1^Department of Radiology, University of Alabama at Birmingham, Birmingham, AL USA

^2^University of Hamburg, Department of Diagnostic and Interventional Radiology and Nuclear Medicine, Universitätsklinikum Hamburg-Eppendorf, Hamburg, Germany

^3^Yale PET Center, Department of Radiology and Biomedical Imaging, Yale University, New Haven, CT, USA

^4^Department of Pediatric Dentistry, University of Alabama at Birmingham, Birmingham, Alabama USA

^5^Department of Medicine, Yale University, New Haven, CT, USA

^6^Department of Pharmacology, Yale University, New Haven, CT, USA

^7^Orion Pharma, Cambridge, UK

**^#^ Correspondence:** Bernadette Marquez-Nostra, 1824 6^th^ Ave. S., Wallace Tumor Institute 310C, Birmingham, AL 35233, USA, (205) 934-2950, bmarqueznostra@uabmc.edu

**Supplementary Materials and Methods:**

*Phage display screening:*

Following previous methods [20,21], the library was first depleted of nonspecific binders by counterselection against an irrelevant protein, neutravidin (ThermoFisher; 31000). The remaining phages then underwent five rounds of positive selection against huCD68. Each round included washing with 1x TBST to remove nonbinders and eluting bound phages with 0.1 M HCl followed by neutralization with 1 M Tris base and amplification in *E. coli*. After the third round of selection, twelve clones were randomly selected and cultured in a deep 96-well plate. The supernatant in each well are the individual phage-Fab clones that were subsequently tested for CD68-specific binding by clonal ELISA. Sanger sequencing was performed by the Keck Sequencing Facility at Yale University to identify the sequences of the enriched binders to huCD68. Each unique Fab gene insert was subcloned into the RH2.2 vector for Fab protein expression (no phage) in *E. coli* BL21 (DE3) (NEB; C2527).

*Optimizing the conjugation of Fab to DFO:*

We optimized the conjugation of DFO to Fab8 by varying the input stoichiometric ratios (4:1, 5:1, 6:1) and analyzing each crude reaction by SEC-HPLC to determine which molar ratio maintained DFO-Fab integrity with the lowest amount of unreacted DFO. Purity (% monomer) was quantified by radio-size exclusion chromatography HPLC (radio-SEC-HPLC) (Agilent 1260 Infinity II) equipped with a flow-ram radioactivity detector (LabLogic) and a TSKgel G3000SWxl column (300 mm x 7.8 mm, Tosoh; 0008541). An isocratic mobile phase consisting of 0.2 M sodium phosphate, 0.2 M sodium chloride and 0.3% reduced Triton X-100 buffer (Millipore; 282103) and a flow rate of 1 mL/min were employed.

*Flow Cytometry:*

THP-1 undifferentiated cells and THP-1 macrophage cells were harvested, filtered to obtain a single-cell suspension, and adjusted to 10,000 cells/µL in ice-cold FACS staining buffer (Invitrogen; 00-4222-26). Fc receptors were blocked using human Fc Block (2.5 µg, BD; 564220) for 15 min at ambient temperature. Then live/dead staining was performed with Zombie R718 viability dye (1:200, BioLegend; 423115) for 15 min in the dark at ambient temperature. Cells were then fixed and permeabilized using BD Cytofix/Cytoperm (BD; 554722) solution for 10 min at 4 °C and washed twice with BD Perm/Wash buffer (BD; 554723). Intracellular staining was performed with 5 µL of 12 µg/mL of FITC mouse anti-human CD68 (BD; 562117) and FITC mouse IgG2b, κ isotype control (BD; 555057) in 100 µL of Perm/Wash buffer for 30 min at 4 °C in the dark. After two washes, cells were resuspended in FACS staining buffer and acquired on a BD LSR Fortessa at the UAB Flow Cytometry and Single Cell Core Facility. Data was analyzed using FlowJo software (FlowJo™ v10.8 Software; BD Life Sciences). To assess huCD68 expression, the proportion of CD68-positive cells in both THP-1 macrophages and THP-1 undifferentiated cells was quantified using FITC based huCD68 staining in live cells. These percentages were then statistically compared using an unpaired t test with Welch’s correction.

**Supplementary Figures:**

**Figure S1: SEC-HPLC of four lead Fabs after protein A purification.** Each Fab eluted at different retention times.

 **Figure S2: Comparison of stoichiometric ratios of DFO to Fab8 (4:1, 5:1, 6:1) via SEC-HPLC at A_220nm_.** SEC-HPLC overlays of the varying ratios, region 1 shows Fab aggregation, region 2 shows Fab monomer, and region 3 shows unreacted DFO. In all conditions, the DFO-Fab monomer had the same areas under the curve (AUC), but the unreacted DFO had the lowest AUC for the 4:1 DFO:Fab ratio. Therefore, increasing excess DFO does not improve the conjugation yield. Rather, the 4:1 DFO:Fab ratio was sufficient to produce DFO-Fab conjugate.

**Figure S3: Radio-iTLC chromatogram of [^89^Zr]Zr-DFO-Fab8 at SA of 7.4 kBq/nmol.** The radiochemical yield was 99%.

**Figure S4:** **Cellular morphology of THP-1 undifferentiated and PMA differentiated THP-1 macrophages under a light microscope**. THP-1 undifferentiated cells, on the left, are in suspension. After treatment with PMA, THP-1 differentiate to macrophage cells and adhere to the flask, as shown on the right. Images were photographed from a light microscope at 10x magnification.

**Figure S5: Gating scheme for flow cytometry analysis of huCD68 expression in THP-1 macrophage.** CD68 expression was evaluated in **A**. THP-1 undifferentiated cells and **B**. THP-1 macrophages. Cells were initially gated to select single-cell populations, followed by exclusion of dead cells using the viability dye. FITC-conjugated anti-human CD68 antibody, isotype control antibody and unstained cells were compared.

**Figure S6: Percentage of huCD68 expression on THP-1 undifferentiated and THP-1 macrophage cells *in vitro*.** Within the live-cell population, CD68 expression was quantified by measuring FITC-huCD68 fluorescence intensity, with thresholds set to identify CD68-positive cells. The expression of huCD68 was significantly upregulated in THP-1 macrophages (25 ± 0.61 %) compared to THP-1 undifferentiated with low huCD68 expression (4.3 ± 0.36 %). Bars represent mean values (n = 3), and error bars represent standard deviations. **** p < 0.0001

**Figure S7: Distribution of huCD68 and BSA plugs. A.** Quantification of antigen plug segmentations over time post-injection. **B.** Plug-to-Muscle Ratios at 1 and 4 h p.i. represented as Standardized Uptake Value Ratio (SUVR). **C.** Target-to-Control SUVR of huCD68-to-BSA over time. **D.** Correlation of normal organs SUV_mean_ from PET imaging to *ex vivo* biodistribution by gamma counting represented as %ID/g. Pearsons r = 0.9702. Simple linear analysis with 90% confidence interval was applied.

**Figure S8:** **Distribution** **of MDA-MB-231 tumors intratumorally injected with THP-1 macrophages. A.** Quantification of tumor segmentations at 1 and 6 h p.i. **B.** Tumor-to-Muscle Ratios at 1 and 4 h p.i. represented as Standardized Uptake Value Ratio (SUVR). **C.** Target-to-Control SUVR of THP-1 macrophage-to-THP-1 undifferentiated over time. **D.** Correlation of normal organs SUV_mean_ from PET imaging to *ex vivo* biodistribution by gamma counting represented as %ID/g. Pearson’s r = 0.9732. Simple linear analysis with 90% confidence interval was applied.

**Figure S9: Histology of THP-1 macrophage injected MDA-MB-231 tumors. A.** H&E images and **B.** IF images of each mouse. Each mouse represented as M1, M2, M3.

**Figure S10: Histology of THP-1 macrophage injected MDA-MB-231 tumors. A.** H&E images and **B.** IF images of each mouse. Each mouse represented as M1, M2, M3

**Supplementary Tables:**

**Table S1: Materials for lysis buffer**

| **Reagent** | **Company; Catalog #** |
| --- | --- |
| Triton-X-100 | ThermoScientific; A16046.AE |
| Benzonase | Sigma-Aldrich; E8263-25KU |
| Magnesium chloride hexahydrate | Sigma-Aldrich; 1.05833.1000 |
| Phenylmethylsulfonyl fluoride (PMSF) | Roche Diagnostic; 1139061001 |
| Lysozyme | American Bio; AB01178-00005 |
| PBS | ThermoScientific; 14190235 |

**Table S2: Ex vivo biodistribution of [^89^Zr]Zr-DFO-Fab8 at 4 h p.i. in BALB/c mice (n = 3) with bilateral antigen plugs.**

| **Tissue** | **%ID/g ± SD** |
| --- | --- |
| Lungs | 4.8 ± 0.78 |
| Liver | 23 ± 1.6 |
| Kidneys | 79 ± 4.5 |
| Heart | 3.4 ± 0.84 |
| Spleen | 15 ± 3.2 |
| huCD68 antigen plug | 9.2 ± 1.8 |
| BSA antigen plug | 6.1 ± 1.1 |
| Blood | 3.0 ± 0.24 |
| Bone | 2.3 ± 0.28 |
| Muscle | 0.84 ± 0.18 |

**Table S3: Ex vivo biodistribution of [^89^Zr]Zr-DFO-Fab8 at 6 h p.i. in NSG mice (n = 3) with bilateral MDA-MB-231 tumors with intratumoral THP-1 macrophages or THP-1 undifferentiated cells.**

| **Tissue** | **%ID/g ± SD** |
| --- | --- |
| Lungs | 6.5 ± 4.1 |
| Liver | 23 ± 1.6 |
| Kidneys | 84 ± 9.0 |
| Heart | 2.3 ± 0.57 |
| Spleen | 15 ± 5.5 |
| THP-1 Macrophages | 1.9 ± 0.63 |
| THP-1 Undifferentiated | 0.77 ± 0.44 |
| Blood | 1.7 ± 1.0 |
| Bone | 2.4 ± 0.42 |
| Muscle | 0.22 ± 0.035 |
